# A macrophage-associated immunosuppressive subtype defined by Lactobacillus/Bifidobacterium-associated host genes characterizes prognosis and the immune microenvironment in hepatocellular carcinoma

**DOI:** 10.3389/fcimb.2026.1918319

**Published:** 2026-10-14

**Authors:** Qimeng Chang, Huarong Mao, Jinfeng Feng, Longhua Rao, Weiguo Tang, Ziping Zhang, Zhiqiu Hu

**Affiliations:** 1 Department of Hepatobiliary and Pancreatic Surgery, Minhang Hospital, Fudan University, Shanghai, China; 2 Key Laboratory of Whole-Period Monitoring and Precise Intervention of Digestive Cancer, Shanghai Municipal Health Commission, Minhang Hospital, Fudan University, Shanghai, China; 3 Institute of Fudan-Minhang Academic Health System, Minhang Hospital, Fudan University, Shanghai, China

**Keywords:** hepatocellular carcinoma, immunosuppression, macrophages, probiotic-response host genes, prognostic model

## Abstract

**Background:**

Lactobacillus/Bifidobacterium-associated host target genes curated in gutMGene v2.0 may reflect expression programs related to the immune microenvironment in hepatocellular carcinoma (HCC), but their significance in prognostic stratification and macrophage states remains unclear.

**Objective:**

To investigate whether operationally defined Lactobacillus/Bifidobacterium-associated host genes (LBHGs) can define prognostic subtypes of HCC and reveal the corresponding immunosuppressive macrophage features.

**Methods:**

LBHGs were operationally defined from Lactobacillus/Bifidobacterium-related host target genes in gutMGene v2.0 human records. TCGA-LIHC was used as the discovery cohort for differential expression, univariate Cox, consensus clustering, LASSO-Cox modeling, and survival analysis; GSE76427 was used for expression pattern, subtype projection, and external validation of the risk model; GSE149614 single-cell data were used to calculate the LBHG score, recluster macrophages, and operationally define LBHG-IMac as the subgroup with the highest median LBHG score and significant upregulation of at least 3 prespecified immunosuppressive/pro-tumor markers, representing an LBHG-high immunosuppressive TAM-like macrophage state, and its top-30 marker signature was quantified in TCGA-LIHC and GSE76427 using ssGSEA with within-cohort z-score standardization.

**Results:**

Among 94 cross-cohort detectable LBHGs, 12 core LBHGs were identified. The LBHG subtype constructed based on these genes showed that LBHG-C2 had worse overall survival (OS) (log-rank P = 0.003). The 7-gene risk model effectively distinguished high- and low-risk patients in TCGA-LIHC (log-rank P = 0.001; the 1-, 3-, and 5-year AUCs were 0.744, 0.711, and 0.736, respectively), and risk score was an independent prognostic factor (HR = 1.94, P = 0.005); this model was externally validated in GSE76427 (log-rank P = 0.006; the AUCs were 0.708, 0.691, and 0.721, respectively). Single-cell analysis identified LBHG-IMac as a LBHG-high immunosuppressive TAM-like macrophage state, which was enriched in primary tumors (P < 0.001). LBHG-IMac score was positively correlated with risk score (both P < 0.001), and the high LBHG-IMac group showed worse OS and positive correlations with the expression of immune checkpoint and immunosuppression-related genes.

**Conclusion:**

The operationally defined LBHG framework can define an adverse prognostic pattern in HCC and are associated with a LBHG-IMac-enriched immunosuppressive microenvironment.

## 1 Introduction

Hepatocellular carcinoma is the major histological type of primary liver cancer, accounting for approximately 80% to 90% of all primary liver cancers, and commonly occurs in the setting of chronic liver disease and liver cirrhosis, with both disease burden and mortality burden being heavy (Llovet et al., 2022). Traditional staging can reflect tumor extent, but it is difficult to fully explain the significantly different survival outcomes among patients at the same stage (Wang et al., 2024). In recent years, research has gradually shifted attention to the gut-liver axis and the tumor immune microenvironment, suggesting that gut microbes and their metabolites can affect hepatic inflammation, metabolic remodeling, and tumor progression through host responses (Silveira et al., 2022). Tumor-associated macrophages are considered an important component of the immunosuppressive microenvironment in hepatocellular carcinoma (HCC), and their heterogeneity is closely related to angiogenesis, invasion and metastasis, immune escape, and treatment response (Zhou et al., 2020; Zheng et al., 2023). Single-cell studies have identified MMP9-positive tumor-associated macrophages (TAMs) related to migration, invasion, and angiogenesis in HCC, indicating that macrophage subtypes may be a key level linking molecular expression patterns and clinical outcomes (Zhang et al., 2024). Existing studies have mostly approached this issue separately from microbial composition, microbial metabolites, or broad immune-related gene sets, and fewer studies have used probiotic-related host target genes as a unified entry point to explain the prognostic heterogeneity of HCC (Lee et al., 2022). Most public tumor cohorts do not contain matched microbiome sequencing data, making it difficult for “microbial changes” to be directly incorporated into transcriptome-based prognostic studies (Roelands et al., 2023). Although another type of study has established various risk models, the candidate genes are derived from broad sources, often lack clear biological boundaries, and rarely answer which cell type the risk signal actually comes from (Qian et al., 2022). gutMGene v2.0 provides manually curated relationships among gut microbes, microbial metabolites, and host target genes, providing a traceable basis for defining Lactobacillus/Bifidobacterium-associated host genes (LBHGs) based on host transcriptomes. Lactobacillus and Bifidobacterium were prespecified because they encompass widely studied probiotic species and strains, providing a focused, hypothesis-driven scope rather than a data-driven selection based on the number of curated records. With respect to Lactobacillus- and Bifidobacterium-related host target genes, whether there are expression patterns with prognostic significance in HCC and whether such patterns correspond to an immunosuppressive macrophage state still lack a systematic answer. Based on this background, this study used LBHGs derived from gutMGene v2.0 as the prespecified candidate source, screened core genes in TCGA-LIHC, constructed subtypes and a prognostic model, completed external validation in GSE76427, and combined GSE149614 single-cell data to localize their cellular basis. The study aimed to clarify whether LBHGs can define a molecular subtype with poor prognosis in HCC, and to reveal whether they are associated with the LBHG-high immunosuppressive TAM-like macrophage state (LBHG-IMac) and related immunosuppressive microenvironmental features.

## 2 Materials and methods

### 2.1 Study design

This study was a retrospective bioinformatics study based on public transcriptomic data, and the data download, curation, and analysis were performed from February 10, 2026 to February 22, 2026. The study included only 4 pre-specified data sources: gutMGene v2.0, TCGA-LIHC, GSE76427 (Grinchuk et al., 2018), and GSE149614 (Lu et al., 2022), without introducing other public cohorts or immune infiltration databases. These sources were prespecified to provide complementary evidence: gutMGene v2.0 for LBHG definition, TCGA-LIHC for discovery and model construction, GSE76427 for independent cross-platform bulk validation, and GSE149614 for single-cell localization; thus, the study was designed as a focused multi-layer analysis rather than exhaustive multi-cohort benchmarking. gutMGene v2.0 is a manually curated database of relationships among gut microbes, microbial metabolites, and host target genes, with humans and mice as the major species, and this study used only the human records therein.

The data of the TCGA-LIHC discovery cohort were obtained from the TCGA-LIHC project in the GDC Data Portal. The project page showed a total of 377 cases; this study downloaded the Gene Expression Quantification data in the Transcriptome Profiling module and the corresponding clinical data, and used GDC Data Release 45.0 as the data version. According to the official GDC description, Release 45.0 was released on December 4, 2025, and the RNA-Seq STAR-Counts files contain raw counts as well as FPKM, FPKM-UQ, and TPM. The GSE76427 external validation cohort was obtained from the GEO database, with GPL10558 as the platform. The official GEO page showed that this cohort included 115 primary HCC tumor tissues and 52 adjacent non-tumor tissues, with a total of 167 samples. The GSE149614 single-cell cohort was also obtained from the GEO database, with GPL24676 as the platform. The official GEO page showed that this cohort included 10 HCC patients, 21 samples, and more than 70,000 single-cell transcriptomes, and provided three processed files: GSE149614_HCC.metadata.updated.txt.gz, GSE149614_HCC.scRNAseq.S71915.count.txt.gz, and GSE149614_HCC.scRNAseq.S71915.normalized.txt.gz. All data were public and de-identified, and this study did not involve additional patient recruitment or intervention; therefore, no additional ethical approval was required. The overall study design workflow is shown in **Figure 1**.

**Figure 1:**
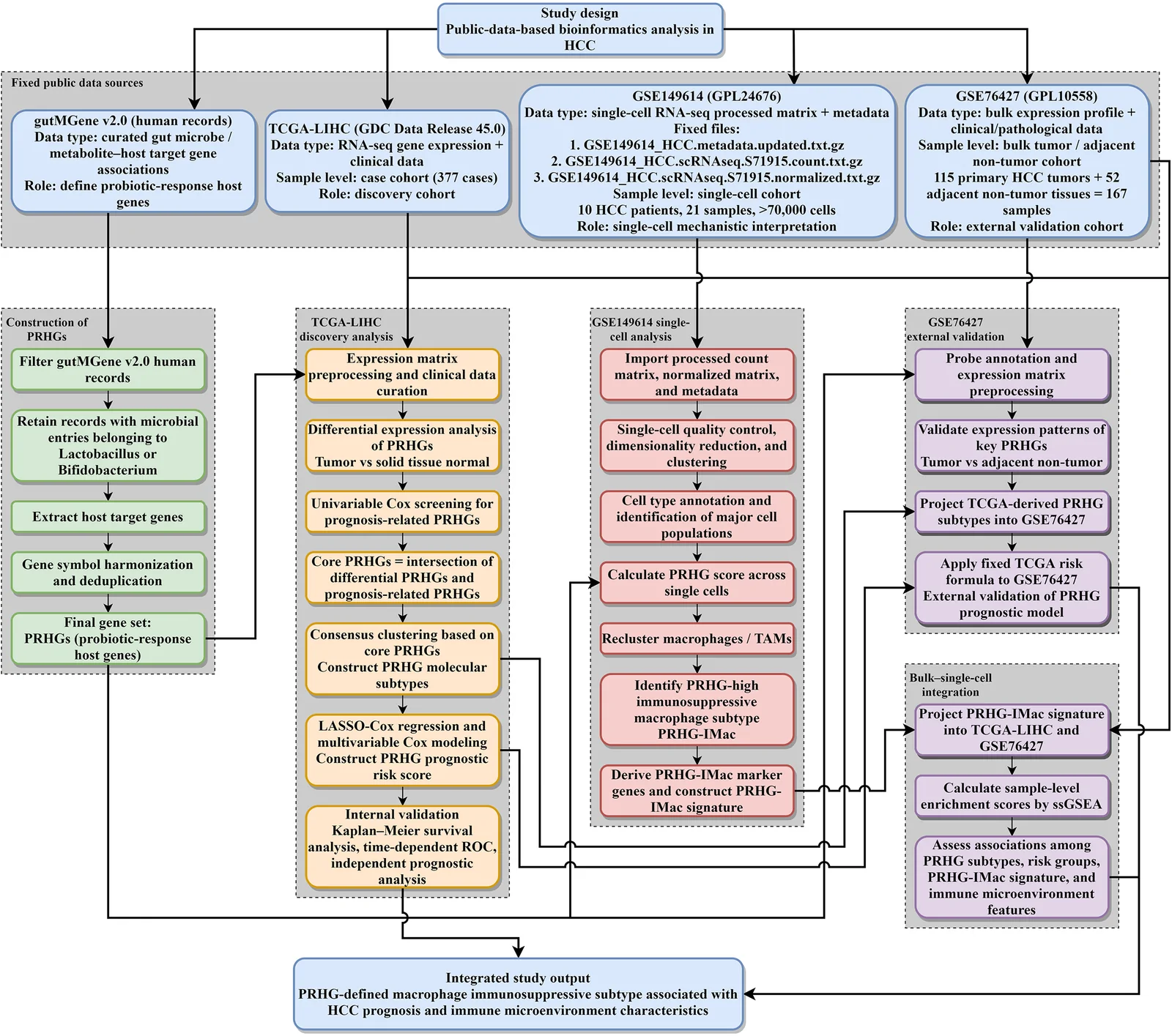
Flow chart of the study design.

### 2.2 Construction of the probiotic-response host gene set

After downloading the human records from gutMGene v2.0, only records with the microbial genus name Lactobacillus or Bifidobacterium were retained according to the pre-specified rules. This hypothesis-driven genus-level restriction was specified before database screening and was not determined by ranking the numbers of curated host target gene records across genera. The screening was based on the original microbial names in the database, without extending to other reclassified genus names, and without secondary screening according to the type of causal/correlative relationship, regulatory direction, or disease label. Only records with a clearly defined host target gene field that could be mapped to standard human gene symbols were included, and deduplication was performed at the gene level to obtain an operationally defined LBHG set. This database-derived designation did not imply liver-specific or intervention-validated probiotic responses in HCC. To ensure the feasibility of cross-platform validation, only LBHGs simultaneously matched in the expression matrices of TCGA-LIHC and GSE76427 were retained in the subsequent bulk cohort analyses.

### 2.3 Analysis of the TCGA-LIHC discovery cohort and model construction

TCGA-LIHC Gene Expression Quantification data and clinical data were downloaded from the GDC Data Portal. The differential expression analysis included primary tumor and solid tissue normal samples with RNA-seq data; the prognostic analysis included cases with primary tumor expression profiles, overall survival time, and survival status. Case-level primary diagnosis and histological annotations were reviewed, and cases annotated as cholangiocarcinoma, combined hepatocellular–cholangiocarcinoma, or other non-HCC malignancies were excluded. Overall survival time was defined by days_to_death or days_to_last_follow_up, and survival status was coded according to vital_status. The original day-level survival times were retained without rounding or discretization for all survival analyses, and Kaplan-Meier estimates were calculated at each observed event time; conversion to months was used only for graphical display. Age, sex, pathological stage, and histological grade were uniformly curated, and stage subcategories were combined into stages I-IV; missing values were not imputed and were handled as complete-case data in the corresponding analyses.

TCGA STAR-Counts raw counts were used for differential expression analysis. Before analysis, low-expression genes with raw counts <10 in more than 80% of samples were removed. DESeq2 was used to compare the expression differences of LBHGs between primary tumor and solid tissue normal samples, and LBHGs with FDR <0.05 after Benjamini-Hochberg correction and |log2FC| >1 were defined as differentially expressed LBHGs. The TCGA TPM matrix was transformed by log2(TPM + 1) and used for subtyping, correlation analysis, and survival modeling. All detectable LBHGs were subjected to univariate Cox regression after gene-level z-score standardization, and LBHGs with P <0.05 after Benjamini-Hochberg correction were defined as prognostic LBHGs; the intersection between these genes and the differentially expressed LBHGs was taken as the core LBHGs.

Based on the standardized expression matrix of the core LBHGs, unsupervised consensus clustering was performed using ConsensusClusterPlus. The number of clusters was set as k=2-6, with 1000 resamplings, 80% resampling proportions for both samples and genes, Pearson correlation distance as the distance metric, and partitioning around medoids as the clustering algorithm. The optimal k was determined jointly by the CDF curve, delta area changes, and consensus matrix stability. PCA was used to assess subtype separation, Kaplan-Meier analysis and the log-rank test were used to compare survival differences between subtypes, and categorical variables were compared using the χ² test or Fisher’s exact test.

The core LBHGs were included in LASSO-Cox regression, and the optimal penalty parameter was determined by 10-fold cross-validation. using the λ1se criterion. A fixed random seed of 20260210 was set before cross-validation to ensure reproducible fold assignment. To assess selection stability, 1,000 stratified 80% subsamples (n = 292 each) were drawn without replacement while preserving the event proportion, using a fixed seed of 20260211. Within each subsample, the 12 core LBHGs were re-standardized and the same 10-fold LASSO-Cox procedure was repeated; gene selection frequency and coefficient-direction consistency were recorded. The genes retained by LASSO were further entered into multivariate Cox regression to construct the final model, and the risk score was calculated as the linear sum of “gene expression × regression coefficient”. To reduce platform differences, the model genes were first z-score standardized within each cohort before calculating the risk score; the TCGA cohort was divided into high- and low-risk groups according to the median risk value. Model performance was evaluated using Kaplan-Meier curves, 1-, 3-, and 5-year time-dependent ROC curves, and Harrell C-index. The independent prognostic value of the risk score was tested by univariate and multivariate Cox regression. The multivariate model pre-specified the inclusion of age, sex, pathological stage, histological grade, and risk score, and the proportional hazards assumption was tested using Schoenfeld residuals.

### 2.4 External validation in GSE76427

The GSE76427 series matrix and accompanying clinical data were downloaded from GEO. This cohort was based on the GPL10558 platform, and the raw data had already been processed by the original study using lumi robust spline normalization (RSN); therefore, the processed RSN expression matrix was used directly. Probes were mapped to gene symbols according to the GPL10558 annotation, and probes without clear symbols were removed; when multiple probes corresponded to the same gene, the median expression value was used to represent gene expression. All 167 samples were used for validation of expression direction, including 115 primary HCC tumors and 52 adjacent non-tumor samples; the intergroup expression trends of key LBHGs were tested using the limma linear model, and FDR <0.05 after Benjamini-Hochberg correction was considered statistically significant. Expression-direction validation was performed within GSE76427, and absolute expression values were not directly compared across the RNA-seq and microarray platforms.

The external prognostic validation included only primary HCC tumor samples with expression profiles and survival endpoint information. Overall survival time was retained at the finest resolution provided in the original clinical annotations, without rounding or discretization to annual time points; Kaplan-Meier estimates were calculated at each observed event time, and any unit conversion was used only for graphical display. During the validation stage, the model genes and regression coefficients established in TCGA were fixed, and genes were not re-screened and the model was not refitted. After z-score standardization of the model genes within GSE76427, the TCGA risk formula was applied to calculate the risk score, and samples were divided into high- and low-risk groups according to the median risk value in GSE76427. The external validation metrics included Kaplan-Meier curves, the log-rank test, 1-, 3-, and 5-year time-dependent ROC curves, and Harrell C-index. To evaluate subtype transferability, core LBHG expression values were standardized gene-wise within TCGA-LIHC and GSE76427 separately. A nearest-centroid classifier was constructed from the standardized TCGA subtype centroids, and the GSE76427 tumor samples were assigned using the correspondingly standardized expression profiles, followed by comparison of their survival and molecular feature differences. The two cohorts were analyzed separately, and no cross-cohort batch correction was applied.

### 2.5 Single-cell transcriptomic analysis of GSE149614

Three fixed processed files of GSE149614 were downloaded from GEO: GSE149614_HCC.metadata.updated.txt.gz, GSE149614_HCC.scRNAseq.S71915.count.txt.gz, and GSE149614_HCC.scRNAseq.S71915.normalized.txt.gz. This cohort included 21 samples and more than 70,000 single-cell transcriptomes from 10 HCC patients, and the sample sources included primary tumors, portal vein tumor thrombi, metastatic lymph nodes, and non-tumor liver tissues. A Seurat object was constructed based on the count matrix, and genes expressed in no fewer than 3 cells were retained; the cell quality control criteria were 200–6000 detected genes and mitochondrial gene proportion <20%. The normalized matrix was used to recheck the distribution of normalized expression and support module scoring and feature visualization, and metadata.updated was used to integrate sample source and the authors’ original cell type information. Harmony was used for correction with sample ID as the batch variable, and UMAP dimensionality reduction and clustering analysis were completed based on the first 30 principal components, with the clustering resolution of the main atlas set at 0.6.

Cell type annotation used the authors’ metadata as the initial reference and was verified in combination with classical marker genes: T cells as CD3D and CD3E, NK cells as NKG7 and KLRD1, B cells as MS4A1 and CD79A, macrophages as LYZ, C1QA, C1QB, and CD68, endothelial cells as PECAM1 and KDR, fibroblasts as COL1A1, COL1A2, and DCN, and malignant hepatocytes as ALB, EPCAM, and KRT8. To be consistent with the bulk primary HCC cohorts, the subsequent inter-tissue statistics of LBHG module scores and the construction of bulk projection signatures were limited to primary tumor and non-tumor liver samples; portal vein tumor thrombus and metastatic lymph node samples were used only for the display of the complete single-cell atlas and the observation of heterogeneity.

Seurat::AddModuleScore was used to calculate the LBHG score of each cell based on the detectable LBHGs in the single-cell matrix (Tirosh et al., 2016). This score represented non-directional relative enrichment of the predefined LBHG set against expression-matched control genes and was distinct from the coefficient-weighted risk score derived from the bulk cohorts. Because the LBHG set was defined independently of GSE149614, cross-cell-type comparisons were used only to localize this predefined signal. Macrophages were then extracted, and highly variable gene screening, Harmony correction, PCA, UMAP, and clustering analysis were reperformed, with the reclustering resolution set at 0.4. To evaluate parameter robustness, reclustering was repeated at resolutions 0.3 and 0.5, and stability was assessed by comparing cell membership and subgroup-specific marker profiles across resolutions. The resolution 0.4 solution was retained because the LBHG-IMac-like population and its marker profile were preserved across adjacent resolutions. Marker genes of each macrophage subgroup were identified using the Wilcoxon rank-sum test, and P <0.05 after Benjamini-Hochberg correction, avg_log2FC >0.25, and pct.1 >0.25 were defined as significant marker genes. The subgroup with the highest median LBHG score and with significant upregulation of at least 3 of MMP9, SPP1, APOE, CD163, MRC1, VSIG4, TGFB1, and IL10 compared with the remaining macrophages was defined as the LBHG-high immunosuppressive macrophage state, namely LBHG-IMac. Statistics involving differences in cell proportions or module scores between tissue sources were all performed at the sample level, with the cell proportions or median LBHG score within each sample first summarized and then compared between groups, so as to avoid single-cell pseudoreplication bias.

### 2.6 Bulk-single-cell integration and immune microenvironment evaluation

Single-cell-derived feature sets were constructed based on the differential expression results between LBHG-IMac and the remaining macrophage subgroups. The screening criteria were P <0.05 after Benjamini-Hochberg correction, avg_log2FC >0.5, and pct.1 >0.25, and mitochondrial genes, ribosomal protein genes, and HLA class genes were removed. Candidate genes were ranked from high to low according to avg_log2FC, and the top 30 genes were used to construct the LBHG-IMac signature. To characterize the HCC immune microenvironment, based on the author-annotated cell groups in the primary tumor and non-tumor liver samples of GSE149614, the top 30 specific marker genes were extracted for macrophage/TAM, CD8+ T cell, NK cell, B cell, endothelial cell, and fibroblast, respectively, to construct the corresponding single-cell-derived feature sets.

In the bulk expression matrices of TCGA-LIHC and GSE76427, the ssGSEA algorithm in the GSVA package was used to calculate the sample-level enrichment scores of the LBHG-IMac signature and the above cell feature sets, and z-score standardization was performed within each cohort. The Wilcoxon rank-sum test was used to compare differences in signature scores among different LBHG subtypes and risk groups, and Spearman correlation analysis was used to evaluate the relationship between the LBHG risk score and the LBHG-IMac signature score. The correlations of the LBHG risk score and LBHG-IMac signature score with the expression of PDCD1, CD274, CTLA4, LAG3, HAVCR2, TIGIT, VSIR, TGFB1, and IL10 were further analyzed, and the survival differences between the high and low LBHG-IMac signature groups were compared according to the cohort median.

### 2.7 Statistical analysis and software environment

All statistical analyses were performed in the R 4.3.2 environment, mainly using the DESeq2, limma, survival, survminer, glmnet, ConsensusClusterPlus, timeROC, Seurat, harmony, and GSVA packages. Comparisons of continuous variables were performed using the Wilcoxon rank-sum test or Kruskal-Wallis test, categorical variables were analyzed using the χ² test or Fisher’s exact test, and correlations were assessed using Spearman rank correlation. Survival analyses were performed using the Kaplan-Meier method, the log-rank test, and Cox proportional hazards regression, and the hazard ratio was expressed as HR and 95% CI. Except that multiple correction had already been explicitly applied in single-cell marker identification and differential expression analysis, the Benjamini-Hochberg method was used in analyses involving parallel testing of multiple genes to control the false discovery rate. All tests were two-sided, and P <0.05 or FDR <0.05 was considered statistically significant.

## 3 Results

### 3.1 Construction of the probiotic-response host gene set

The primary HCC tumor samples used for prognostic analysis included 365 cases from TCGA-LIHC and 115 cases from GSE76427. Both cohorts were dominated by males, early to intermediate pathological stages, and G2-G3 grades. The numbers of death events in TCGA-LIHC and GSE76427 were 127 and 43, respectively, and the median follow-up times were 22.8 months and 40.7 months, respectively, supporting the subsequent overall survival analysis and external validation (**Table 1**).

**Table 1:** Clinicopathological characteristics of the TCGA-LIHC discovery cohort and the GSE76427 validation cohort.

Variable level	TCGA-LIHC	GSE76427
Samples for prognostic analysis	365	115
Age (years)	61 (53, 69)	64 (56, 71)
Male	250 (68.49%)	83 (72.17%)
Female	115 (31.51%)	32 (27.83%)
Pathological stage		
I	169 (46.30%)	52 (45.22%)
II	83 (22.74%)	30 (26.09%)
III	78 (21.37%)	22 (19.13%)
IV	5 (1.37%)	3 (2.61%)
Unknown	30 (8.22%)	8 (6.96%)
Histological grade		
G1	52 (14.25%)	18 (15.65%)
G2	171 (46.85%)	54 (46.96%)
G3	118 (32.33%)	34 (29.57%)
G4	13 (3.56%)	3 (2.61%)
Unknown	11 (3.01%)	6 (5.22%)
Alive	238 (65.21%)	72 (62.61%)
Dead	127 (34.79%)	43 (37.39%)
Follow-up time (months)	22.8 (13.1, 44.6)	40.7 (24.3, 65.8)

This table included only primary HCC tumor samples used for prognostic analysis; Unknown indicates missing or unspecified information in the original data; continuous variables are presented as Median (Q1, Q3), and categorical variables are presented as n (%); this table presents descriptive statistics, and no P values were set.

### 3.2 Screening and subtyping of core LBHGs in TCGA-LIHC

Among the 94 cross-cohort detectable LBHGs, the differential expression analysis in TCGA-LIHC identified 36 differentially expressed LBHGs, including 24 upregulated genes and 12 downregulated genes (**Figure 2A**). Univariate Cox regression identified 15 prognostic LBHGs, including 10 with HR > 1 and 5 with HR < 1 (**Figure 2B**). After taking the intersection of the two sets, 12 core LBHGs were obtained, among which MMP9, SPP1, CXCL8, CCL20, PLAUR, LCN2, SERPINE1, and LGALS3 were upregulated risk genes, whereas HNF4A, SLC10A1, APOA1, and CYP3A4 were downregulated protective genes, and these genes were used for subsequent molecular subtyping and prognostic model construction (**Figure 2C**).

**Figure 2:**
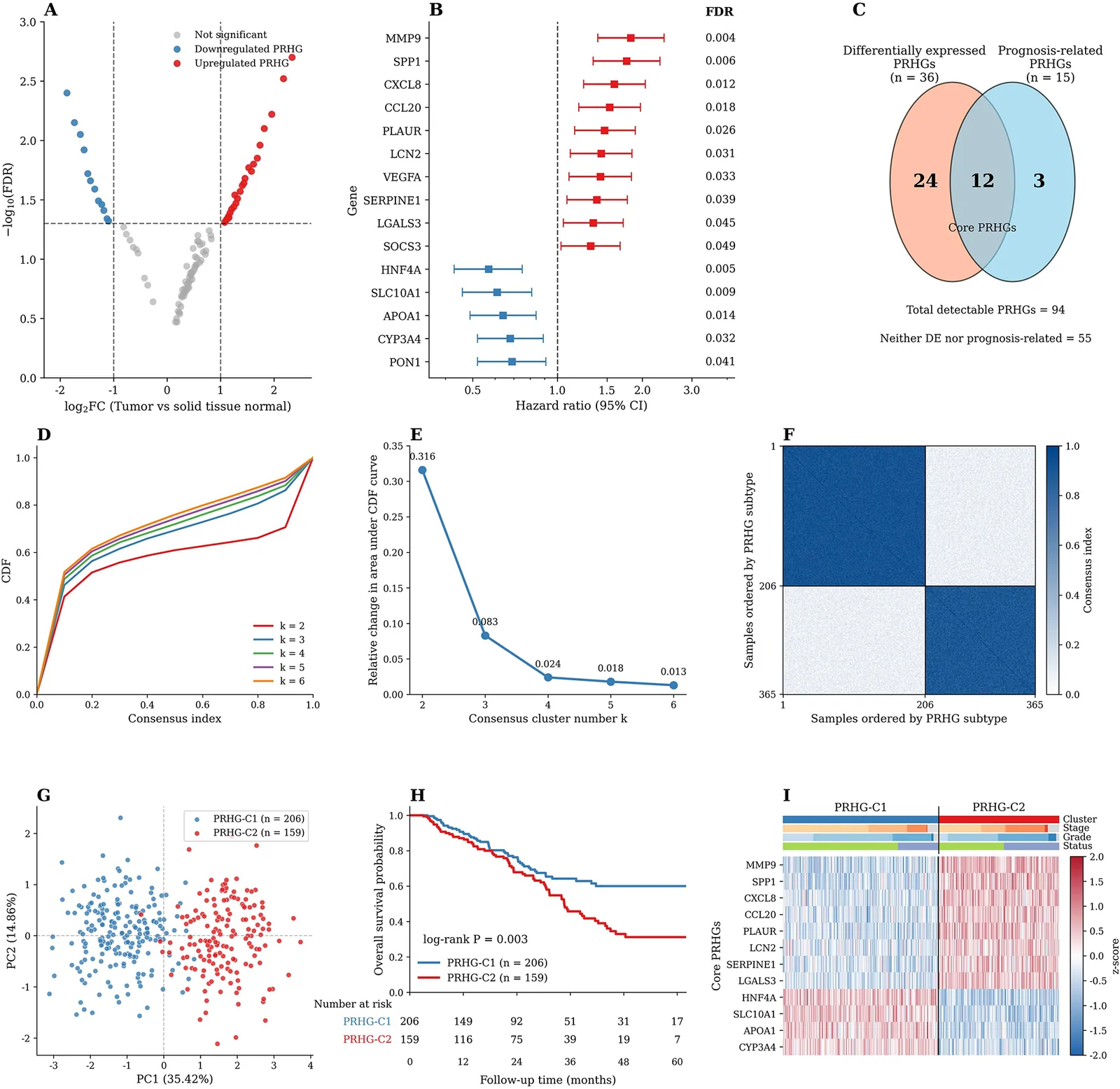
Screening, molecular subtyping, and clinical significance of core LBHGs in TCGA-LIHC. **(A)** Volcano plot of differential expression of LBHGs between tumor tissues and solid tissue normal samples; **(B)** Forest plot of prognostic LBHGs screened by univariate Cox regression; **(C)** Intersection analysis of differentially expressed LBHGs and prognostic LBHGs; **(D)** Consensus clustering CDF curves; **(E)** Delta area curves for different cluster numbers k; **(F)** Consensus matrix at k = 2; **(G)** PCA scatter plot based on the expression profile of core LBHGs; **(H)** Kaplan-Meier overall survival curves of LBHG-C1 and LBHG-C2; **(I)** Expression heatmap of core LBHGs and clinical annotations.

Based on the consensus clustering analysis of the 12 core LBHGs, k = 2 was determined as the optimal subtype, and 365 TCGA-LIHC patients were divided into 206 cases in the LBHG-C1 group and 159 cases in the LBHG-C2 group (**Figures 2D–F**). The PCA scatter plot showed that the two groups were well separated in the expression space of the core LBHGs, and PC1 and PC2 explained 35.42% and 14.86% of the variance, respectively (**Figure 2G**). Survival analysis showed that the proportion of death events was higher in the LBHG-C2 group than in the LBHG-C1 group, and overall survival was significantly worse in the LBHG-C2 group (log-rank P = 0.003); the median OS of the LBHG-C2 group was 34.7 months, whereas the median OS was not reached in the LBHG-C1 group (**Figure 2H**). The expression heatmap showed that LBHG-C2 was mainly characterized by increased expression of upregulated risk LBHGs and decreased expression of downregulated protective LBHGs (**Figure 2I**).

Differential expression analysis was performed using DESeq2, and prognostic analysis was performed using univariate Cox proportional hazards regression; FDR was corrected by the Benjamini-Hochberg method; subtyping was constructed based on the log2(TPM + 1) expression matrix of the 12 core LBHGs, and the heatmap expression values were z-score standardized by gene. LBHGs, LBHGs; HR, hazard ratio; CI, confidence interval; FDR, false discovery rate; CDF, cumulative distribution function; PCA, principal component analysis; OS, overall survival.

### 3.3 Construction of the LBHG prognostic risk model and validation in the discovery cohort

LASSO-Cox regression screened 7 model genes from the 12 core LBHGs, including 5 risk genes (MMP9, SPP1, CXCL8, PLAUR, and SERPINE1) and 2 protective genes (HNF4A and SLC10A1) (**Figures 3A, B**). In the resampling-based stability analysis, selection frequencies were 93.40% for MMP9, 88.70% for SPP1, 75.80% for CXCL8, 80.60% for PLAUR, 71.30% for SERPINE1, 91.10% for HNF4A, and 84.90% for SLC10A1; each of the five non-model core LBHGs was selected in <50.00% of subsamples. Among subsamples in which each model gene was selected, coefficient-direction consistency ranged from 97.90% to 100.00%, supporting the robustness of the selected genes. According to the final risk score, 365 TCGA-LIHC patients were divided into 183 cases in the low-risk group and 182 cases in the high-risk group; the proportion of death events was higher in the high-risk group than in the low-risk group (48.90% vs 20.77%), and the high-risk group showed increased expression of risk genes and decreased expression of protective genes (**Figures 3C–E**). Cross-tabulation showed that the proportion of high-risk patients was higher in LBHG-C2 than in LBHG-C1 (100/159, 62.89% vs 82/206, 39.81%; Pearson χ² = 19.133, P < 0.001), indicating a significant but incomplete association between the two stratification approaches. Kaplan-Meier analysis showed that the overall survival of the high-risk group was significantly worse than that of the low-risk group (log-rank P = 0.001), among which the median OS of the high-risk group was 31.8 months, whereas the median OS was not reached in the low-risk group. Time-dependent ROC analysis showed that the AUCs of this model for predicting 1-, 3-, and 5-year OS were 0.744, 0.711, and 0.736, respectively, indicating that it had good prognostic discrimination ability in the discovery cohort (**Figures 3F, G**). The multivariate Cox results showed that the HRs of genes with positive coefficients in the final LBHG prognostic risk model were all > 1, the HRs of genes with negative coefficients were all < 1, and all 7 model genes reached statistical significance (all P < 0.05) (**Table 2**). In the TCGA-LIHC cohort, univariate Cox analysis showed that pathological stage III/IV, histological grade G3, and the LBHG risk score were associated with worse OS (all P < 0.05). Multivariate Cox analysis further showed that the LBHG risk score and pathological stage III remained independent prognostic factors for OS (all P < 0.05) (**Table 3**).

**Figure 3:**
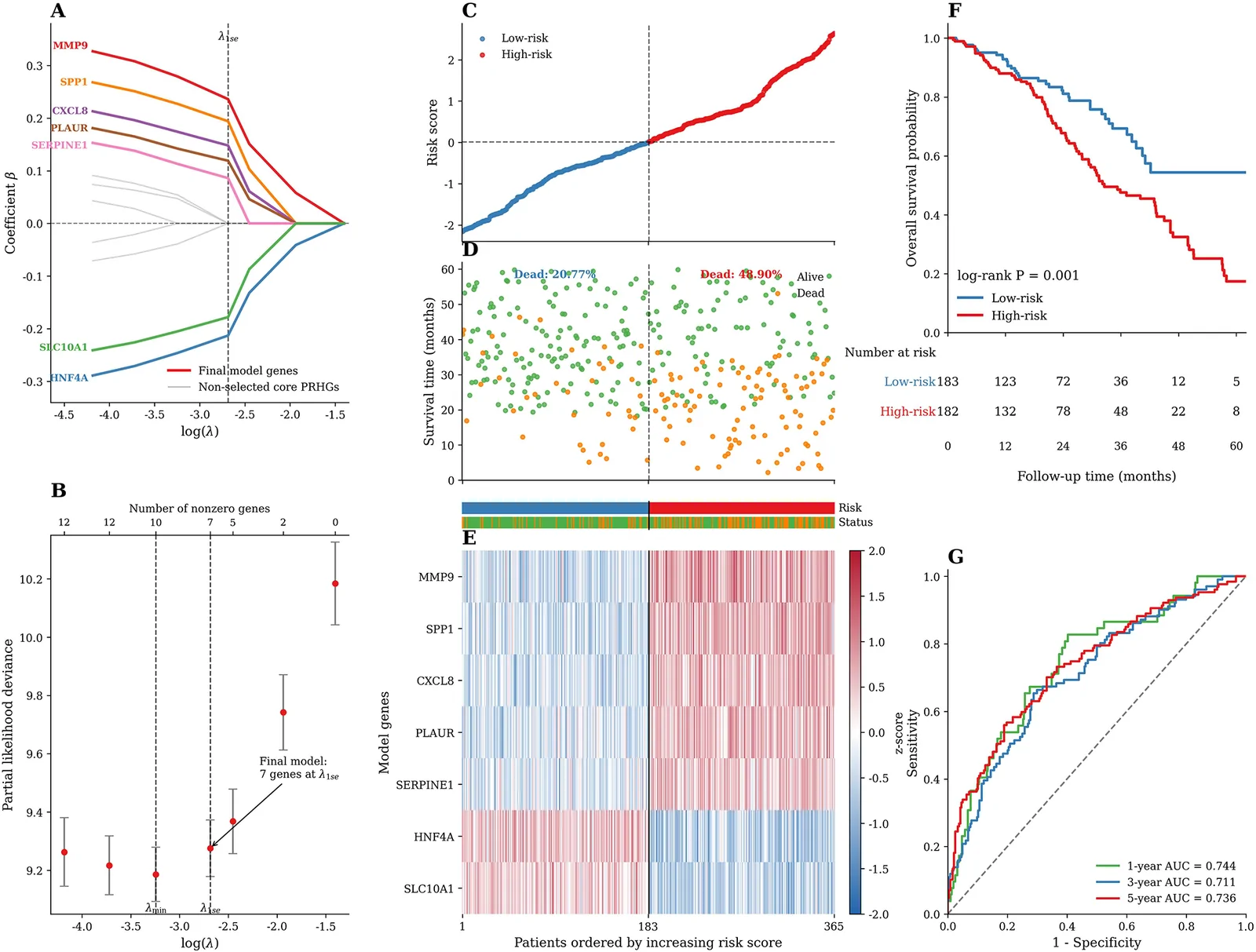
Construction and internal validation of the LBHG prognostic risk model in TCGA-LIHC. **(A)** Coefficient profile plot of LASSO-Cox regression; **(B)** 10-fold cross-validation to determine the optimal penalty parameter λ; **(C)** Distribution of patients ranked by risk score; **(D)** Distribution of survival status ranked by risk score; **(E)** Heatmap of model gene expression; **(F)** Kaplan-Meier overall survival curves of the high- and low-risk groups; **(G)** Time-dependent ROC curves for 1-, 3-, and 5-year overall survival.

**Table 2:** Model genes included in the LBHG prognostic risk model and multivariate Cox regression results.

Gene symbol	β	HR (95% CI)	P
MMP9	0.309	1.36 (1.14–1.63)	0.001
SPP1	0.266	1.30 (1.10–1.55)	0.003
CXCL8	0.188	1.21 (1.03–1.41)	0.020
PLAUR	0.227	1.25 (1.06–1.48)	0.008
SERPINE1	0.173	1.19 (1.02–1.39)	0.029
HNF4A	−0.284	0.75 (0.64–0.89)	0.001
SLC10A1	−0.239	0.79 (0.67–0.93)	0.004

β indicates the regression coefficient of the final multivariate Cox model; HR indicates the relative risk corresponding to each 1 standard deviation increase in the expression of the model gene; the expression values of all genes were z-score standardized within the cohort before modeling.

**Table 3:** Cox regression analysis of OS in the TCGA-LIHC cohort.

Variable	Univariate, HR (95% CI)	P	Multivariate, HR (95% CI)	P
Age, continuous variable	1.01 (0.99–1.02)	0.191	1.01 (0.99–1.03)	0.325
Sex, male vs female	1.12 (0.78–1.60)	0.536	1.08 (0.74–1.57)	0.688
Pathological stage, II vs I	1.48 (0.92–2.37)	0.104	1.26 (0.76–2.09)	0.370
Pathological stage, III vs I	2.22 (1.39–3.56)	0.001	1.86 (1.13–3.06)	0.015
Pathological stage, IV vs I	3.68 (1.14–11.86)	0.029	2.19 (0.65–7.38)	0.206
Histological grade, G2 vs G1	1.31 (0.72–2.38)	0.376	1.12 (0.61–2.05)	0.714
Histological grade, G3 vs G1	1.83 (1.08–3.11)	0.025	1.34 (0.78–2.29)	0.287
Histological grade, G4 vs G1	2.51 (0.96–6.54)	0.060	1.42 (0.52–3.88)	0.494
risk score, continuous variable	2.36 (1.39–4.01)	0.001	1.94 (1.22–3.08)	0.005

The outcome variable was overall survival (OS); the multivariate Cox model included age, sex, pathological stage, histological grade, and risk score; the reference categories were female, pathological stage I, and histological grade G1; Unknown cases were not included in the corresponding model, and the multivariate analysis used complete-case data (n = 327); the proportional hazards assumption test showed global P = 0.427.

The expression values of the model genes were z-score standardized within the cohort and then entered into the Cox coefficients to calculate the risk score, and the high- and low-risk groups were divided according to the median risk score of the TCGA-LIHC cohort. LBHGs, LBHGs; LASSO, least absolute shrinkage and selection operator; OS, overall survival; ROC, receiver operating characteristic; AUC, area under the curve.

### 3.4 Validation of LBHG expression, subtypes, and the risk model in GSE76427

In the external validation cohort GSE76427, the expression directions of the 7 model genes were consistent with the findings in TCGA-LIHC. MMP9, SPP1, CXCL8, PLAUR, and SERPINE1 were upregulated in HCC tumor tissues, whereas HNF4A and SLC10A1 were downregulated in tumor tissues, and all differences reached statistical significance (all FDR < 0.05) (**Figure 4A**). In GSE76427, based on the projection of the TCGA-LIHC subtype centroids, 115 primary HCC tumor samples were classified into 64 cases in the projected LBHG-C1 group and 51 cases in the projected LBHG-C2 group. The projected subtypes were used to assess transferability of the 12-core-LBHG subtype structure, whereas the risk groups evaluated the external performance of the fixed 7-gene prognostic model. The projected LBHG-C2 group showed increased expression of risk LBHGs and decreased expression of protective LBHGs, and its overall survival was significantly worse than that of the projected LBHG-C1 group (log-rank P = 0.021) (**Figures 4B, C**). After fixed application of the TCGA-LIHC-derived 7-gene risk formula, the GSE76427 cohort was divided into 58 cases in the low-risk group and 57 cases in the high-risk group. Cross-tabulation showed that high-risk patients were more frequent in projected LBHG-C2 than in projected LBHG-C1 (32/51, 62.75% vs 25/64, 39.06%; Pearson χ² = 6.368, P = 0.012), indicating a significant but incomplete association between the two stratification approaches. The proportion of death events was higher in the high-risk group than in the low-risk group (52.63% vs 22.41%), and the high-risk group showed increased expression of risk genes and decreased expression of protective genes. Kaplan-Meier analysis showed that OS was significantly worse in the high-risk group (log-rank P = 0.006); time-dependent ROC analysis showed that the 1-, 3-, and 5-year AUCs were 0.708, 0.691, and 0.721, respectively (**Figures 4D–H**).

**Figure 4:**
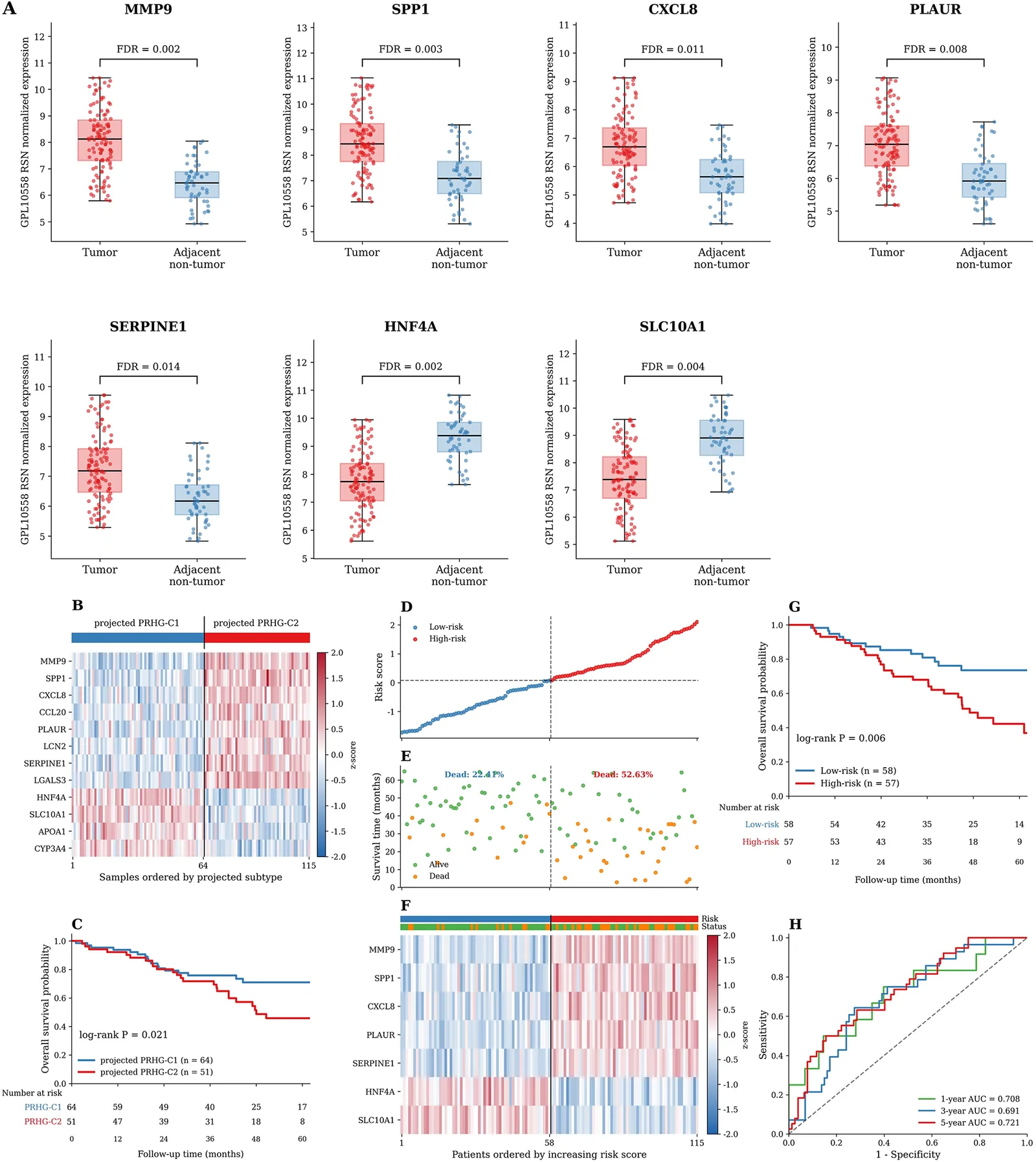
External validation of LBHG subtypes and the risk model in GSE76427. **(A)** Expression differences of the 7 model LBHGs in Tumor and adjacent non-tumor samples; **(B)** Heatmap of core LBHG expression based on the projection of TCGA-LIHC subtype centroids; **(C)** Kaplan-Meier overall survival curves of projected LBHG-C1 and projected LBHG-C2; **(D)** Distribution of patients ranked by risk score; **(E)** Distribution of survival status ranked by risk score; **(F)** Heatmap of model gene expression; **(G)** Kaplan-Meier overall survival curves of the high- and low-risk groups; **(H)** Time-dependent ROC curves for 1-, 3-, and 5-year overall survival.

Intergroup comparisons in panel A were performed using the limma linear model, and FDR was corrected by the Benjamini-Hochberg method; subtype projection in GSE76427 was completed based on the core LBHG expression centroids of LBHG-C1 and LBHG-C2 in TCGA-LIHC; the risk score was calculated using the TCGA-LIHC-derived 7-gene model and Cox coefficients, and the high- and low-risk groups were divided according to the median risk score of the GSE76427 cohort. LBHGs, LBHGs; FDR, false discovery rate; OS, overall survival; ROC, receiver operating characteristic; AUC, area under the curve.

### 3.5 Single-cell analysis of the LBHG-IMac state and its immune associations

A total of 71,843 cells were included in the GSE149614 single-cell atlas, mainly including malignant hepatocytes, T cells, macrophages, endothelial cells, fibroblasts, NK cells, and B cells (**Figures 5A, B**). The LBHG score showed a heterogeneous distribution across different cell types, among which macrophages had the highest overall LBHG score, suggesting that the LBHG signal was mainly concentrated in the macrophage population (**Figures 5C, D**).

**Figure 5:**
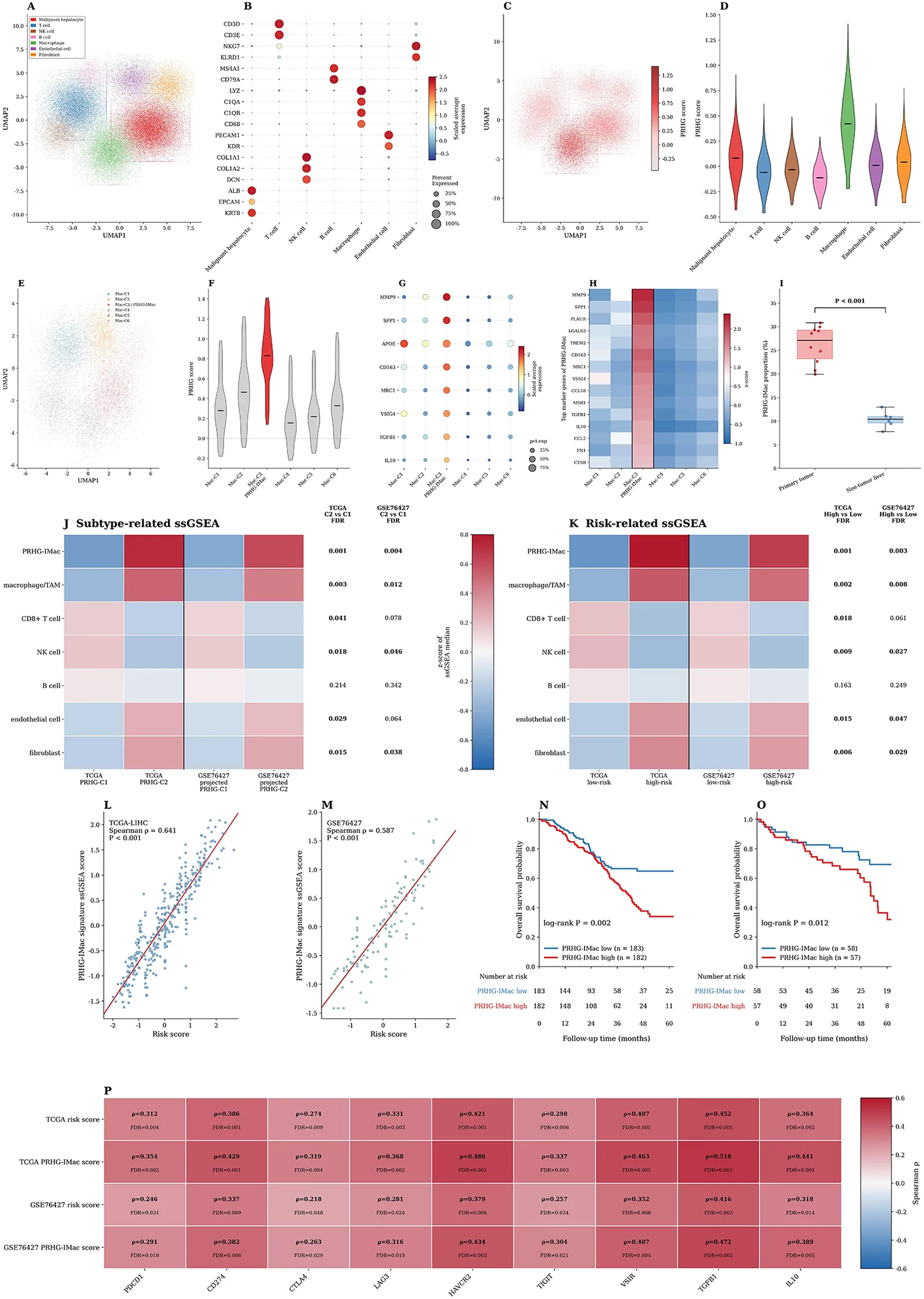
Single-cell analysis of the LBHG-IMac state and its immune microenvironment and prognostic associations. **(A)** UMAP plot of major cell types in GSE149614; **(B)** Dot plot of cell type marker genes; **(C)** UMAP distribution of LBHG score; **(D)** Distribution of LBHG score across different cell types; **(E)** UMAP plot of macrophage reclustering; **(F)** Distribution of LBHG score across macrophage subgroups; **(G)** Expression of immunosuppression/pro-tumor-related genes; **(H)** Heatmap of LBHG-IMac marker genes; **(I)** Sample-level comparison of the proportion of LBHG-IMac between primary tumor and non-tumor liver samples using the Wilcoxon rank-sum test, with samples rather than individual cells as the statistical units; **(J)** ssGSEA heatmap related to LBHG subtypes; **(K)** ssGSEA heatmap related to risk stratification; **(L, M)** Correlation between LBHG-IMac score and risk score in TCGA-LIHC and GSE76427; **(N, O)** Overall survival curves stratified by LBHG-IMac score in TCGA-LIHC and GSE76427; **(P)** Correlation heatmap of risk score, LBHG-IMac score, and immune checkpoint and immunosuppression-related genes.

In GSE149614, 12,781 macrophages were reclustered into 6 subgroups, among which Mac-C3 contained 2,397 cells, accounting for 18.75% of all macrophages (**Figure 5E**). Across resolutions 0.3–0.5, a corresponding LBHG-IMac-like population was consistently recovered, with preserved enrichment of its defining markers, supporting the robustness of the resolution 0.4 solution. Mac-C3 had the highest median LBHG score and significantly upregulated immunosuppression/pro-tumor-related genes such as MMP9, SPP1, CD163, MRC1, VSIG4, TGFB1, and IL10, and was therefore designated LBHG-IMac, representing a LBHG-high immunosuppressive TAM-like macrophage state (**Figures 5F, G**). The marker gene heatmap of LBHG-IMac showed that this subgroup highly expressed genes such as MMP9, SPP1, PLAUR, LGALS3, TREM2, CD163, MRC1, and VSIG4 (**Figure 5H**). Using samples rather than individual cells as the statistical units, sample-level analysis showed that the proportion of LBHG-IMac was higher in primary tumor samples than in non-tumor liver tissues (Wilcoxon rank-sum test, P < 0.001) (**Figure 5I**).

Projection of the single-cell-derived feature sets showed that LBHG-C2, and projected LBHG-C2 in GSE76427, as well as the high-risk group, all showed increased LBHG-IMac, macrophage/TAM, and fibroblast features, together with decreased NK cell features in both TCGA-LIHC and GSE76427 (all FDR < 0.05). No stable differences were observed in B cell features across subtyping and risk stratification in either cohort (**Figures 5J, K**).

LBHG-IMac score was positively correlated with risk score in both TCGA-LIHC and GSE76427 (both P < 0.001) (**Figures 5L, M**). After grouping by the median LBHG-IMac score, the high LBHG-IMac group showed worse OS in both cohorts (both log-rank P < 0.05) (**Figures 5N, O**). The correlation heatmap showed that risk score and LBHG-IMac score were overall positively correlated with the displayed immune checkpoint and immunosuppression-related genes, and all correlations reached statistical significance (FDR < 0.05) (**Figure 5P**).

LBHG score was calculated using Seurat::AddModuleScore; LBHG-IMac was operationally designated as the macrophage subgroup with the highest LBHG score and significant upregulation of immunosuppression/pro-tumor-related genes; ssGSEA scores were z-score standardized within each bulk cohort. Panel I used samples as the statistical unit; intergroup comparisons were performed using the Wilcoxon rank-sum test, correlations were analyzed using Spearman analysis, survival comparisons were performed using the log-rank test, and FDR was corrected by the Benjamini-Hochberg method. LBHGs, LBHGs; LBHG-IMac, LBHG-high immunosuppressive macrophage state; UMAP, uniform manifold approximation and projection; ssGSEA, single-sample gene set enrichment analysis; FDR, false discovery rate.

## 4 Discussion

The core genes further distilled from the cross-cohort detectable LBHGs constituted an expression framework capable of distinguishing the molecular heterogeneity of HCC. The LBHG subtypes established on this basis were not merely statistical groupings, but biological states corresponding to clearly separated clinical outcomes. LBHG-C2 was characterized by overall increased risk genes and overall decreased protective genes, together with worse survival, suggesting that Lactobacillus- and Bifidobacterium-related host target genes were sufficient to define a tumor phenotype with poor prognosis. Its underlying basis was more likely the coupling of pro-inflammatory, pro-invasive, and dedifferentiation processes. MMP9, SPP1, PLAUR, SERPINE1, and LGALS3 are mostly involved in extracellular matrix remodeling, infiltrative migration, and the formation of an immunosuppressive microenvironment, whereas CXCL8, CCL20, and LCN2 point to enhanced inflammatory chemotaxis and stress responses (Volponi et al., 2022). The downregulation of HNF4A, SLC10A1, APOA1, and CYP3A4 suggested impaired mature hepatocyte function and damaged liver-specific metabolic programs (Kotulkar et al., 2023). This combination was closer to the inflammation-driven and differentiation-loss phenotype commonly seen in advanced HCC, rather than random fluctuations of isolated genes (Song et al., 2022). LBHGs were derived from Lactobacillus- and Bifidobacterium-related host target genes in gutMGene and represented a traceable definition of host response; they were not equivalent to the abundance of probiotics in patients, and could not be extrapolated as the therapeutic effect of probiotics. Previous studies have mostly discussed HCC heterogeneity from microbial composition, metabolite changes, or broad immune gene sets, and fewer studies have established prognostic subtypes using probiotic-related host target genes as the entry point (Li et al., 2023a; Yu et al., 2021). The subtype direction obtained after integrating microbe-related host target genes with tumor transcriptomes was consistent with the existing understanding of enhanced inflammation, matrix remodeling, and hepatocyte dedifferentiation in HCC, and laid the molecular foundation for further localizing prognostic differences to an immunosuppressive macrophage state.

The risk model composed of 7 LBHGs could stably distinguish survival risk in the discovery cohort and still maintained a consistent stratification direction and acceptable discriminatory ability in the external cohort, indicating that it was not an incidental result formed by fitting a single cohort. The risk score retained independent prognostic value after adjustment for age, sex, pathological stage, and histological grade, suggesting that it reflected molecular risk information beyond traditional clinical indicators, rather than a simple repetition of tumor burden (Li et al., 2020). Pathological stage still had independent significance, further indicating that this model was more suitable as a supplementary stratification tool outside the staging system, rather than a substitute for clinical staging. The 5 risk genes in the model jointly pointed to activation of inflammatory chemotaxis, extracellular matrix remodeling, angiogenesis, and invasive migration, whereas the 2 protective genes corresponded to a relatively intact hepatocyte differentiation and transport-metabolism phenotype; therefore, the risk score was closer to a composite expression index in which pro-tumor inflammation enhancement and liver function loss coexisted (Li et al., 2023a; Aoki et al., 2025). Previous HCC prognostic models were mostly derived from broad sources and often lacked a clear biological starting point or strict external validation (Schnabl et al., 2025). Here, the candidate genes were restricted to Lactobacillus- and Bifidobacterium-related host target genes, and the formula was fixed for cross-platform validation, giving the model clearer source constraints and interpretability. The subsequent single-cell and bulk integration results further anchored the high-risk state to the background of LBHG-IMac and macrophage/TAM enrichment, allowing this model to have not only prognostic stratification significance, but also a cellular ecological interpretation at the level of the immune microenvironment. This made it closer to the biological process of HCC progression than a purely statistical model.

Single-cell-level localization provided a cellular context for the LBHG-associated expression pattern observed in the high-risk group. The single-cell LBHG score, a non-directional module-enrichment measure distinct from the bulk risk score, was not uniformly distributed across all cell populations, but was mainly concentrated in macrophages, suggesting that the LBHG signal in the bulk cohorts contained a significant myeloid component (Li et al., 2023b). The LBHG-IMac state identified after macrophage reclustering had both the highest LBHG module enrichment and immunosuppressive and pro-tumor features such as MMP9, SPP1, CD163, MRC1, VSIG4, TGFB1, and IL10, and was enriched in primary tumors. This indicated that the LBHG expression pattern corresponded to a TAM-related immune niche (Sharma et al., 2020). After projecting its features back to the two bulk cohorts, both LBHG-C2 and the high-risk group were accompanied by increased LBHG-IMac, macrophage/TAM, and fibroblast features and decreased NK cell features, LBHG-IMac score was stably positively correlated with risk score, and the high LBHG-IMac state also corresponded to worse survival, suggesting that poor prognosis was associated with a microenvironmental context of enhanced myeloid immunosuppression and weakened antitumor cytotoxicity (Jin et al., 2024). MMP9 and SPP1 point to matrix degradation, angiogenesis, and invasive migration, CD163, MRC1, and VSIG4 reflect an immunosuppression-biased TAM state, and TGFB1 and IL10 support the activation of immunosuppressive factors (Quaranta et al., 2024). This marker profile overlaps with established immunosuppressive TAM programs (Jin et al., 2024; Quaranta et al., 2024); therefore, LBHG-IMac should be interpreted as a LBHG-high TAM-like state rather than a novel macrophage lineage. The overall positive correlations of risk score and LBHG-IMac score with multiple immune checkpoints and immunosuppression-related genes supported the co-occurrence of the high-risk expression pattern and the immunosuppressive network, but it could not yet be inferred from this that LBHG-IMac directly drove checkpoint upregulation (Hsieh and Chuang, 2025). Previous single-cell studies have suggested the presence of MMP9-positive TAMs and marked macrophage heterogeneity in HCC (Wu et al., 2024). On this basis, the present results further connected the framework of Lactobacillus- and Bifidobacterium-related host target genes with the immunosuppressive macrophage state, thereby supporting the localization of the LBHG prognostic signal to an immunosuppressive TAM-like state.

Limitations: This study was a retrospective analysis based on public transcriptomic data, and the conclusions obtained were expression associations and could not replace mechanistic evidence. LBHGs were derived from the operational definition of Lactobacillus- and Bifidobacterium-related host target genes in gutMGene, and the study itself did not include microbiome sequencing, probiotic intake information, or paired samples before and after intervention; therefore, it could not be used to determine the true abundance changes of probiotics in HCC patients, nor could it be used to infer their direct regulation of LBHG-IMac. The LBHG-defined groups were not systematically compared with established HCC molecular classifications, and their relationship to existing taxonomies remains undetermined. The discovery cohort and the validation cohort used RNA-seq and microarray platforms, respectively. Although platform differences had been controlled through within-cohort standardization and fixed model coefficients, measurement error, missing clinical information, and differences in sample composition could still affect model extrapolation. Moreover, the risk model was externally validated in only one independent bulk cohort and showed moderate discrimination; its generalizability across populations, etiologies, and platforms requires confirmation in additional independent cohorts. The single-cell part relied on only one cohort, and the definition of LBHG-IMac was based on module scores, clustering results, and marker gene selection; more single-cell data, spatial transcriptomics, protein-level validation by immunohistochemistry or multiplex immunofluorescence, and functional assays of macrophage polarization and T-cell suppression are still needed to validate its spatial localization and biological function. Immune microenvironment evaluation relied on the projection of the single-cell-derived signature in the bulk cohorts, ssGSEA reflected relative enrichment rather than true cell proportions, and the correlations of risk score and LBHG-IMac score with immune checkpoint expression could only indicate co-occurrence relationships and could not be used to establish a causal chain.

## 5 Conclusion

The LBHG expression framework can identify patient groups with poor prognosis and immunosuppressive features in hepatocellular carcinoma. The risk model established based on this framework showed consistent prognostic stratification in one independent external cohort. Single-cell analysis suggests that the LBHG high-risk state is associated with the tumor-enriched LBHG-IMac TAM-like macrophage state, which is accompanied by enhanced TAM features, weakened NK cell features, and increased immune checkpoint-related signals. The association between LBHGs and LBHG-IMac can serve as a candidate molecular-cellular framework for examining potential associations among gutMGene-derived Lactobacillus/Bifidobacterium-associated host expression patterns, prognostic differences in hepatocellular carcinoma, and immunosuppressive microenvironmental features.

## Data Availability

The datasets presented in this study are publicly available. TCGA-LIHC data are available from the Genomic Data Commons (GDC) Data Portal under the TCGA-LIHC project. The GSE76427 and GSE149614 datasets are available from the NCBI Gene Expression Omnibus (GEO) under accession numbers GSE76427 and GSE149614, respectively. The Lactobacillus/Bifidobacterium-associated host target gene information used in this study was obtained from the publicly available gutMGene v2.0 database.
